# Molecular Mechanisms and Potential Antiviral Strategies of Liquid–Liquid Phase Separation during Coronavirus Infection

**DOI:** 10.3390/biom14070748

**Published:** 2024-06-24

**Authors:** Ying Wang, Liying Zhou, Xiaohan Wu, Shixing Yang, Xiaochun Wang, Quan Shen, Yuwei Liu, Wen Zhang, Likai Ji

**Affiliations:** School of Medicine, Jiangsu University, Zhenjiang 212013, China; 2212313049@stmail.ujs.edu.cn (Y.W.); 2212213056@stmail.ujs.edu.cn (L.Z.); 2212213101@stmail.ujs.edu.cn (X.W.); 1000004113@ujs.edu.cn (S.Y.); jdwxc@ujs.edu.cn (X.W.); shenquan@ujs.edu.cn (Q.S.); liuyuwei@ujs.edu.cn (Y.L.)

**Keywords:** SARS-CoV-2, nucleocapsid protein, liquid–liquid phase separation, innate immunity

## Abstract

Highly pathogenic coronaviruses have caused significant outbreaks in humans and animals, posing a serious threat to public health. The rapid global spread of severe acute respiratory syndrome coronavirus-2 (SARS-CoV-2) has resulted in millions of infections and deaths. However, the mechanisms through which coronaviruses evade a host’s antiviral immune system are not well understood. Liquid–liquid phase separation (LLPS) is a recently discovered mechanism that can selectively isolate cellular components to regulate biological processes, including host antiviral innate immune signal transduction pathways. This review focuses on the mechanism of coronavirus-induced LLPS and strategies for utilizing LLPS to evade the host antiviral innate immune response, along with potential antiviral therapeutic drugs and methods. It aims to provide a more comprehensive understanding and novel insights for researchers studying LLPS induced by pandemic viruses.

## 1. Introduction

Cellular biological processes with complex regulatory mechanisms are often localized to specific regions, encompassing various membrane-bound organelles and non-membrane-bound organelles. These distinct regions play crucial roles in maintaining cellular homeostasis and facilitating various biological activities by segregating and compartmentalizing the cellular components [1]. Such regulation of cellular processes is fundamental to the understanding of cellular biology and has far-reaching implications in various fields of study. Unlike membranous organelles, membraneless organelles (MLOs) rely on liquid–liquid phase separation (LLPS) to assemble proteins and nucleic acids [2]. Examples of MLOs include Cajal and promyelocytic leukemia (PML) bodies in the nucleus and processing bodies (PBs) and stress granules (SGs) in the cytoplasm [3]. LLPS can selectively polymerize or segregate specific cytoplasmic components and plays an important role in regulating biological processes. The host’s natural immune system encodes several pathogen pattern recognition receptors (PRRs) [4]. These receptors recognize the pathogenic molecular pattern of the pathogen, initiating the production of interferons and cytokines with antiviral and immunomodulatory functions through a complex intracellular signaling process. They serve as the first barrier against the invasion of pathogenic microorganisms [5].

Coronaviruses (CoVs) form a highly diverse pathogenic virus family inducing human and animal diseases. Low-pathogenic human CoVs (HCoVs), such as HCoV-229E, HCoV-OC43, HCoV-NL63, and HCoV-HKU1, cause the common cold. High-pathogenic HCoVs, such as SARS-CoV-1, MERV-CoV, and SARS-CoV-2, can develop into severe, life-threatening respiratory pathologies and lung injuries [6]. In the past, they have caused major widespread outbreaks. Moreover, CoVs that infect livestock and poultry species are an important veterinary and economic concern. Recent studies have shown that CoV infection can modulate LLPS in host cells, influencing viral replication and immune evasion mechanisms [7,8]. Hence, this review focuses on the most recent advancements in basic and applied research in this field, with the goal of enhancing our understanding of the pathogenic mechanisms of coronaviruses and facilitating the development of effective antiviral strategies.

## 2. Coronavirus Genome and Its Life Cycle in Infected Cells

**Figure 1:**
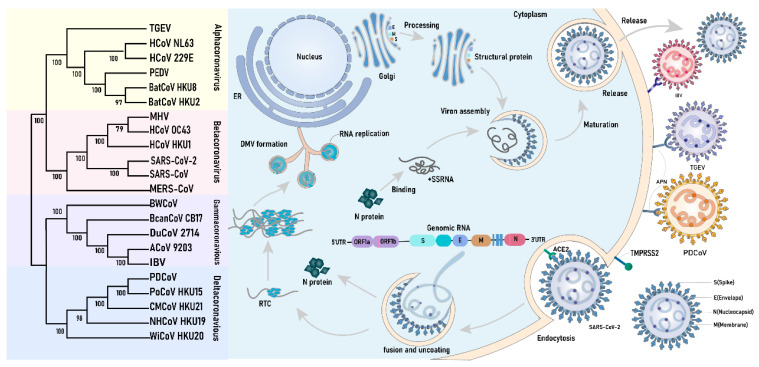
The classes of Coronavirus and their life cycle. Coronaviruses are classified into four distinct genera: *α*-CoV, *β*-CoV, *γ*-CoV, and *δ*-CoV. The life cycle of the four genera of coronaviruses is essentially similar, except for differences in entry into the cellular receptor (e.g., SARS-CoV-2 spiny protein binding to ACE2, TGEV and PDCoV binding to porcine APN). Coronavirus spiking proteins bind to the cellular receptor and enter the intracellular space by plasma membrane fusion in the presence of cytosolic proteases. Viral N proteins and RNA are released, and the ORF1a/b proteins are translated and processed into non-structural proteins to form RTCs and DMVs, with the RNA replicating in the DMVs and eventually extruding from the membrane pores. Structural proteins are translated and then transported to the Golgi apparatus via ERGIC. These newly synthesized viral structural proteins (N, E, M, and S) and genomic +ssRNA are reassembled to form progeny virus, which eventually exits the cell.

CoVs are a cluster of enveloped positive-stranded RNA viruses within *Coronaviridae*. CoVs are classified into four distinct genera: *alphacoronavirus* (*α*-CoV), *betacoronavirus* (*β*-CoV), *gammacoronavirus* (*γ*-CoV), and *deltacoronavirus* (*δ*-CoV). The genome of CoVs is about 26k~32k bp, encoding various essential proteins for viral replication and propagation [9]. Although the genome length and the number of encoded proteins vary among *Coronavirus* genera, they all share a similar basic structure and function [10] (Figure 1). The CoVs ORF1a/b encode 15 or 16 non-structural proteins (Nsps), while the other ORFs encode accessory and structural proteins [11] (Figure 1). The four main structural proteins of coronaviruses are the spike (S), envelope (E), membrane (M), and nucleocapsid (N) proteins (Figure 1).

CoVs invade susceptible cells and complete the assembly and release of progeny viruses within the cells (Figure 1). They primarily infect cells by binding their trimeric S glycoproteins to the special cell-surface receptors, such as aminopeptidase N (APN) and angiotensin-converting enzyme 2 (ACE2) [12,13]. The cleavage of the S protein by the host serine protease TMPRSS2 triggers the fusion of the coronavirus with the plasma membrane, allowing it to enter the cell through endocytosis [14]. Moreover, the heparan sulfate proteoglycan, C-type lectin, and heat shock homologous protein 70 (HSC70) can also serve as cofactors for the viral invasion of cells [15]. Upon viral entry, the fusion of the viral and endosomal membranes facilitates viral uncoating and the release of the viral genome into the host cytoplasm. Subsequently, it is translated into polyproteins pp1a and pp1ab, which are further processed into non-structural proteins [16]. These Nsps can participate in the construction of the replication and transcription complex (RTC) and reshape the cell membrane to form double-membraned replication organelles (DMVs). Viral RNA replication occurs within the DMVs, and the newly synthesized viral RNA can enter the cytoplasm through pores in the DMV membrane. CoVs’ M and E proteins initiate the process of viral assembly via facilitating the formation of the endoplasmic reticulum–Golgi intermediate compartment (ERGIC). CoV N proteins are primarily distributed in the cytoplasm, where they package the viral genome into ribonucleoprotein complexes (RNPs) with hexahedral or tetrahedral shapes. Additionally, they bind viral genome RNA to form phase-separated condensates at the DMVs and ERGIC [17]. The dense protein–nucleic acid compartments can protect the host by isolating host cell proteins from the host immunity system and concentrate viral components to increase the efficiency of viral replication. Upon the maturation of virion assembly, its vesicles merge with the plasma membrane, resulting in the release of the virus through a cytolytic process [18]. Recent studies have also suggested the involvement of lysosomal transport in the release of viruses from infected cells [19].

## 3. LLPS Plays an Essential Role in the Host Antiviral Innate Immune Response

**Figure 2:**
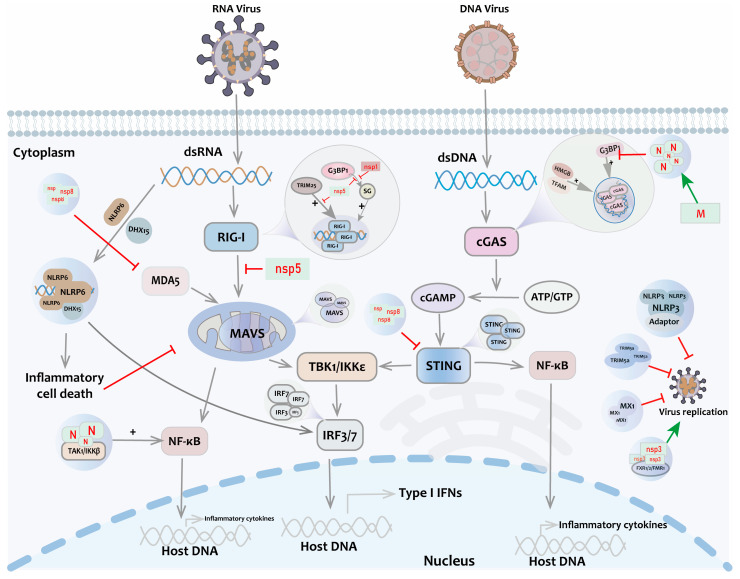
LLPS in the innate immune pathway. After stimulation by viral RNA/DNA, key immune molecules such as RIG-I, MAVS, cGAS, IRF3, and STING undergo LLPS to activate IFN signaling. Positive regulation of antiviral response is also present with TRIM25, NEMO, TFAM, HMGB, etc. Antiviral factors such as TRIM5a, MX1, NLRP3, NLRP6, etc., form liquid-like condensates and mediate antiviral immune response. At the same time, non-structural proteins of coronaviruses such as Nsp5 and Nsp1 participate in the negative regulation of antiviral responses.

Nucleic acids and proteins produced by the invasion of pathogenic microorganisms are primarily recognized by PRRs on the cell surface or within the cell. This recognition triggers the expression of type I interferon (IFN-I) through a signaling cascade reaction. IFN-I plays a crucial role in antiviral and immunomodulatory functions [20]. Currently, PRRs identified in mammals include intracellular DNA sensors, RIG-I-like receptors (RLRs), Toll-like receptors (TLRs), and NOD-like receptors (nucleotide-binding domain and leucine-rich repeat-containing receptors (NLRs) [21]. In recent years, it has been found that PRRs and signal transduction proteins can aggregate by initiating LLPS to regulate the antiviral response (Figure 2).

The cyclic GMP-AMP synthase (cGAS), a nucleotidyltransferase enzyme, recognizes free double-stranded DNA (dsDNA) in cells, initiating a natural immune response. The activity and function of cGAS are regulated by several mechanisms. cGAS interacts with the heterologous nuclear RNA-binding protein G3BP1, leading to the recruitment of cGAS monomers in the cytoplasm to induce LLPS. This complex enhances cGAS enzyme activity by improving the efficiency of cGAS binding to form homodimers during phase separation [22]. When cells are infected by DNA viruses or experience cellular damage, HMGB and TFAM recognize free dsDNA released into the cytoplasm. This recognition prompts the transformation of the DNA structure into a U-shaped form that easily binds to cGAS homodimers [23]. When dsDNA-cGAS binds to form a complex, more cGAS molecules are rapidly recruited to bind free DNA regions, forming a liquid–liquid phase-separated structure known as the cGAS-DNA gel. This structure enhances both the binding of cGAS to DNA and the enzyme-catalyzing activity of cGAS, leading to the production of large amounts of cyclic GMP-AMPP (cGAMP) from GTP and ATP in the cytoplasm [24]. cGAMP is an important second messenger that activates the stimulator of interferon gene (STING) and their downstream signaling pathways on the endoplasmic reticulum membrane. This activation leads to the production of interferon and other cytokines, which trigger natural immune responses against viral infections and cellular damage. In host cells, STING form droplet-like structures via LLPS, which can serve as signaling platforms to facilitate the interaction of STINGs with other signaling molecules such as TBK1 and IRF3 [25]. Hence, LLPS is required for cGAS/STING-induced interferon activation and is critical for host defense against DNA virus infection (Figure 2).

Similar to the DNA sensor cGAS, the retinoic acid-inducible gene-I (RIG-I), a cytosolic PRR for viral RNA, binds to viral RNA, forming LLPS structures named RIG-I gels [24]. TRIM25 catalyzes the activation of K63-linked polyubiquitination of the RIG-I CARD structural domain, which can enhance the binding of the RIG-I PRY/SPRY structural domains to viral RNA, leading to the initiation of LLPS [26]. The G3BP1-mediated formation of stress granules (SGs) can recruit RIG-I and MAVS, enhancing the recognition of viral double-stranded RNA (dsRNA) and activating downstream signaling pathways [27]. Hence, RIG-I gels promote the aggregation and activation of downstream signaling molecules to trigger the production of interferon and enhance the antiviral immune response.

PRRs recruit the serine/threonine protein kinases TBK1 and IKKε to activate the IFN regulatory factors IRF3 and IRF7. The phosphorylated IRF3/7 directly transactivates IFN-stimulated response element (ISRE) in IFN-I promoters to induce IFN production. IRF3-binding ISRE DNA forms liquid-like droplets both in vitro and in vivo. Sirtuin 1 (SIRT1) is the crucial deacetylase for forming IRF3/7 nuclear puncta to improve innate antiviral immunity in the host [28]. SIRT1 interacts with IRF3/7 and then catalyzes deacetylation at Lys39/77 and Lys45/92 of their DBD domains.

NLR family domain-containing PY structure protein 3 (NLRP3) is a significant component of the inflammasome, which can be assembled through LLPS, thereby enhancing the production of interferons and inflammatory factors [29]. The NLRP3 inflammasome can also undergo LLPS with adaptor proteins in the TLR signaling pathway. By forming droplet structures, these proteins create specific subcellular formations within the cell, which can modulate immune signaling and the initiation of apoptosis [25]. NLRP6 recognizes and binds to a variety of pathogen molecules, such as double-stranded RNA (dsRNA) and bacterial LTA. When NLRP6 interacts with these pathogen molecules, the LLPS phenomenon occurs, leading to the formation of droplet-like structures that recruit other signaling molecules, such as DHX15, to participate in the activation of the interferon pathway [30]. The NACHT structural domain of NLRP6 is an important region for dsRNA droplet formation. Upon NLRP6 binding to ASC proteins, the droplets undergo “maturation” and “solidification”, which promote the assembly and activation of inflammatory vesicles [30]. However, it is still unclear how dsRNA or other ligands induce or promote the LLPS mechanism of NLRP6. Further studies are needed to investigate whether other signaling molecules can participate in the LLPS process of NLRP6.

In addition to PRRs, the functions of some other antiviral factors are also associated with LLPS. Myxovirus resistance protein 1 (MX1) is an antiviral factor that can limit viral replication by forming LLPS structures [31]. The triple helix structural domain-containing protein 5a (TRIM5a) can also undergo LLPS, but its connection with antiviral capacity requires further investigation [32]. LLPS also plays an important role in regulating the NF-κB signaling pathway during viral infections. The droplets formed by LLPS can also recruit the TAK1 and IKK complexes, which are key kinases of the NF-κB signaling pathway (Figure 2). This recruitment enhances the activation of NF-κB, promoting the inflammatory response and immune responses [33]. In addition, the ubiquitin-binding (NUB) domain and zinc finger (ZF) domain of NEMO (NF-κB activator) in the NF-κB signaling pathway contribute to its K63-linked polyubiquitination modification. This occurs in LLPS-regulated NF-κB activation and inflammatory factor production [34]. Moreover, LLPS also promotes the assembly and regulation of transcription factors involved in immune regulation in host cells [35].

## 4. The Mechanism of Coronavirus-Induced LLPS

**Figure 3:**
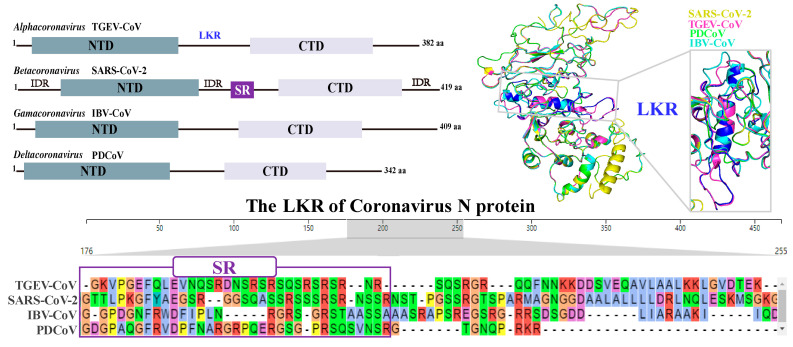
The conserved domains of different CoV-encoded N proteins are included, NTD and CTD, and the LKR. In particular, SARS-CoV-2 N protein contains a special serine-rich (SR) region, which has large differences among different coronavirus genera. Moreover, SARS-CoV-2 N protein contains three intrinsically disordered regions (IDRs), including an N-terminal IDR, a center IDR, and a C-terminal IDR.

Phase separation is a common occurrence in the life cycle of HCoVs [36]. The mechanism of coronavirus-induced LLPS involves the interaction between viral N proteins and RNA. LLPS is a shared intrinsic property among seven homologous N proteins from different HCoVs, undergoing phase separation under distinct in vitro conditions [37]. The initiation of LLPS by a major CoV N protein depends on its RNA binding and post-translational modification. It has been shown that CoV N proteins can form droplets by binding to RNA through charge interactions. The shape and size of these droplets depend on the concentration ratio of the CoV N protein to RNA and the length of RNA [38]. CoV N proteins possess two relatively conserved N-terminal structural domains (NTDs), a C-terminal structural domain (CTD) and a central linker region (LKR) [39] (Figure 3). In particular, the SARS-CoV-2 N protein contains three intrinsically disordered regions (IDRs), including an N-terminal IDR, a center IDR, and a C-terminal IDR [40,41]. Recently, the N-terminal IDR was confirmed as a necessary region for SARS-CoV-2 N protein aggregation causing LLPS [41]. The CTD can self-bind to form oligomers, and this self-binding contributes to the overall stability of the SARS-CoV-2 N protein. There is a conserved positively charged pocket in the CTD structure, which has been suggested to be the RNA binding site. The CTD-RNA binding of the SARS-CoV-2 N protein can mediate LLPS induction [42]. The LKR of SARS-CoV-2 N protein contains a special serine/arginine (SR) region, which can recognize and bind RNA (Figure 3). It mainly consists of a four-stranded antiparallel *β*-folded core sub-structural domain with a protruding β-hairpin consisting of basic amino acid residues. The RNA binding site is located on the positively charged pocket that connects the *β*-hairpin to the core structure [43]. Moreover, the N-terminal region of the SARS-CoV-2 N protein is more flexible than the N protein structures of other coronaviruses. This flexibility may aid in its adaptation to the higher-order structure of the viral RNA genome [44]. These characteristics of the SARS-CoV-2 N protein that are conducive to RNA binding need to be further explored when promoting the induction of LLPS in the cytoplasm. Additionally, the SARS-CoV-2 N protein is SUMOylated by SUMO E3 ligase TRIM28, which efficiently mediates its ability in homo-oligomerization, RNA association, and LLPS [45]. The SARS-CoV-2 N protein depends on its C-terminal IDR to interact with viral M proteins, which is important for the assembling and packaging of virus particles [46]. The phosphorylation ability of SR-rich regions enables participation in the regulation of LLPS behaviors, such as the tendency of RNA-induced N proteins to segregate and the viscosity of condensates [47]. It remains to be further studied whether other types of modifications, except ubiquitination and phosphorylation, affect the CoV N protein’s binding of RNA to cause LLPS.

The beginning of LLPS by the CoV N protein also depends on suitable environmental variables. It was found that the low critical solution phase transition temperature (LCST) must be below physiological values. At ~20 °C, no droplets were found even in the presence of RNA. This indicates that 20 °C < LCST < 37 °C. pH and salt concentration also affect the behavior of LLPS. LLPS increases with decreasing pH values, likely because a low pH allows for partial deprotonation of positively charged N proteins. In turn, this increases the surface tension and promotes hydrophobic-driven segregation. Alternatively, low ionic strength facilitates contact between the negatively charged RNA backbone and the positively charged N protein. At the same time, a low ionic strength may also enhance RNA–RNA repulsion and weaken all hydrophobic interactions [48]. In addition, adenosine triphosphate (ATP) can also modulate the process of LLPS. ATP induces LLPS at low concentrations and dissociates LLPS at high concentrations by specifically binding to arginine/lysine residues in the RNA-binding domains of the N proteins. S2m, a 32-oligomer stem-loop II nucleic acid motif derived from SARS-CoV-1, shares highly overlapping binding sites in the folded NTD and IDR of N proteins and interacts with ATP in the same way to regulate the LLPS of N proteins [49].

Recently, it has been found that CoV Nsps and M proteins can also affect LLPS. The SARS-CoV-2 M protein independently induces N protein phase separation, contributing to recruiting the stress granule protein G3BP1 [46]. SARS-CoV-2 Nsp8, an important non-structural protein, is involved in the formation of the viral genome replication complex [50]. Recent studies have confirmed that SARS-CoV-2 Nsp8 is involved in the process of LLPS. This phase separation phenomenon is regulated by the oligomerization state of Nsp8 and physicochemical changes in the environment. In extracellular assays, Nsp8 dimers specifically form a separate liquid phase at low salt concentrations, whereas Nsp8 tetramers form solid aggregates at low salt concentrations but can be transformed into liquid gels upon the addition of RNA. In intracellular assays, SARS-CoV-2 Nsp8s form gels in the cytoplasm and rapidly fuse or divide [51]. Moreover, both SARS-CoV Nsp1 and MERS-CoV Nsp3 can bind to RNA and participate in LLPS initiation. The multimerization and interaction of CoV Nsps with RNA may be a key factor in the regulation of LLPS [52]. Host fragile X-related (FXR) family proteins (FXR1/FXR2/FMR1) are recruited by SARS-CoV-2 Nsp3 to cluster viral DMVs via LLPS for efficient viral replication [37].

## 5. Mechanisms by Which Coronavirus-Regulated Phase Separation Interferes with IFN-I Production

In SARS-CoV-2 infection, LLPS leads to the coalescence of biomolecules (e.g., proteins or nucleic acids) that form dense droplet-like phases, which is an important process for viral replication assembly and dissemination [53]. In addition, CoVs evade the host immune response by interfering with the production of IFN-I through the modulation of LLPS (Figure 2).

In virus-infected cells, stress granules (SGs) sequester viral RNAs and proteins, thereby disrupting the viral replication cycle [54]. Some CoV-encoded Nsps, such as Nsp5 and Nsp1, can affect LLPS and inhibit SG formation, thereby helping the virus to evade the host’s antiviral response. CoV Nsp5 is the main viral protease for cleaving ORF1a/b to form a variety of viral non-structural proteins and host proteins. SARS-CoV-2 Nsp5 interacts with G3BP1 to inhibit its aggregation and subsequent SG formation. Notably, SARS-CoV-2 Nsp5 does not directly cleave G3BP1 through its protease activity but prefers to competitively bind to the SG component and interrupt G3BP1-triggered aggregate formation [54]. Further studies concluded that Nsp5 not only disrupted the RIG-I-MAVS interaction but also impaired the RIG-I-TRIM25 interaction. Thus, Nsp5 may target multiple steps in the RLR pathway to inhibit the signal cascade. Overexpression of SARS-CoV-2 Nsp5 also leads to reduced levels of TBK1 phosphorylation downstream of RIG-I activation and the inhibition of IRF3 phosphorylation, which is a key step in IFN expression [55]. The Nsp1 protein of SARS-CoV-2 inhibits the phosphorylation of SGs by repressing eIF2α phosphorylation, affects SG formation by inhibiting eIF2α phosphorylation and affecting SG nuclear aggregation and composition, and binds to RNA to form a complex that promotes LLPS [56].

SARS-CoV-2 infection leads to the accumulation of released mitochondrial DNA (mtDNA), which in turn triggers cGAS to activate IFN-I signaling. As a countermeasure, SARS-CoV-2 N proteins can bind to G3BP1 proteins to form droplets and impede the formation of the cGAS–G3BP1 complex. This action reduces the recognition of DNA by cGAS and the LLPS process, ultimately inhibiting cGAS-mediated activation of the IFN signaling pathway [22]. On the other hand, N proteins can form functional membrane-free organelles upon binding to viral RNA. They can also recruit TAK1 and IKK complexes, which promote the activation of NF-κB. In turn, the activated NF-κB leads to the production of inflammatory factors, such as IL-6 [33]. SARS-CoV-2 regulates the hyperactivation of NF-κB through the LLPS of N proteins, which may be responsible for the aberrant inflammatory response and cytokine storms [33]. Moreover, The NLRP6 protein plays a crucial regulatory role during coronavirus infection. When cells are infected by CoVs, NLRP6 binds to viral RNA and forms phase-separated droplets. These droplet-like structures facilitate the interaction of NLRP6 with other proteins (e.g., ASC), leading to the activation of NLRP6 inflammatory vesicles. The activated NLRP6 inflammasome also contributes to the activation of cysteine protease 1 (caspase-1), which triggers inflammatory cell death (pyroptosis), subsequently inhibiting IFN-I production [30]. In summary, these mechanisms may help us better understand the abnormal inflammatory response caused by SARS-CoV-2 infection and provide new therapeutic targets for the treatment of COVID-19.

## 6. Potential Strategies for LLPS-Based Treatment of Coronaviruses

**Figure 4:**
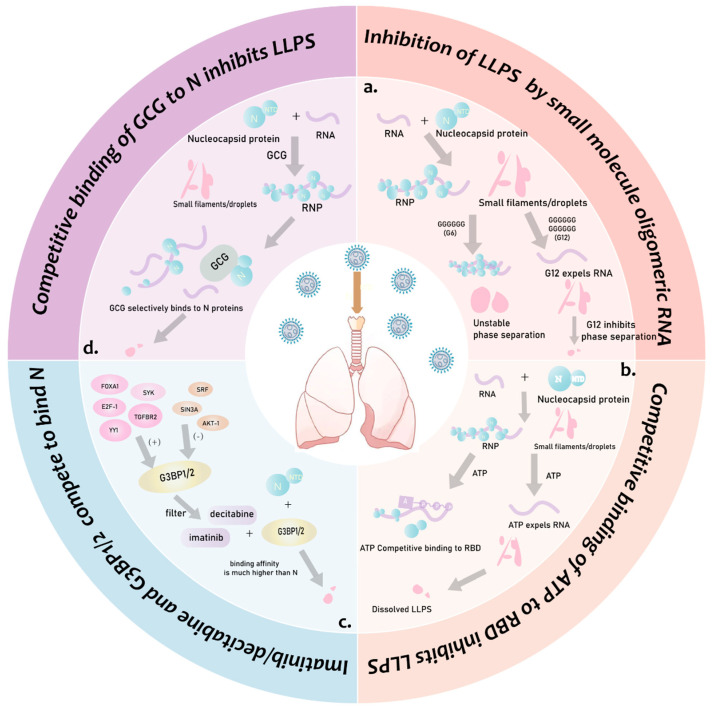
Summary of LLPS-based therapeutic strategies. (**a**). Small-molecule oligo-gua nosine RNA affects LLPS: G12 inhibits liquid-phase separation by expelling RNA from separated droplets, and G6 forms unstable liquid-phase structures. (**b**). Competition for ATP binding to the RBD and expulsion of RNA from the liquid-phase structure inhibits LLPS. (**c**). Screening of genes regulating G3BP1/2 resulted in inhibition of IFN induction by screening for imatinib and decitabine to inhibit LLPS by taking advantage of their much higher binding to G3BP1/2 than the coronavirus N protein property to inhibit IFN induction. (**d**). GCG selectively binds N proteins to inhibit LLPS.

LLPS is an emerging cell biology phenomenon that has recently been found to play an important role in the SARS-CoV-2 nucleocapsid protein. The SARS-CoV-2 nucleocapsid protein achieves LLPS by binding to various viral/host cell nucleic acids [52]. Therefore, LLPS-based therapeutic strategies may be one of the potential approaches to treat coronavirus infections (Figure 4). LLPS modulators can inhibit viral replication through several mechanisms. First, they may interfere with the unpacking process of viral nucleic acids, thus preventing viral replication. Second, they may interfere with the assembly and packaging of viral proteins, thereby preventing viral transmission [53].

Interference with the nucleocapsid protein and RNA interaction involves identifying and designing small-molecule compounds that can disrupt the interaction between N proteins and RNA. This approach has the potential to hinder the initiation of LLPS, thus impeding the replication and transmission of the virus. Oligomeric RNA has been found to inhibit the interaction of CoV N proteins with RNA, interfering with virus replication and transmission [57]. Gallocatechin gallate (GCG), a polyphenol in green tea, interferes with N-RNA binding in other viruses. It also disrupts CoV N-induced LLPS and inhibits SARS-CoV-2 replication with a starting concentration of 6–8 μM [57]. In addition, some studies have shown that Perylene derivatives with specific molecular structures can significantly enhance the liquid–liquid phase separation (LLPS) phenomenon of N-RNA. However, there is a lack of in-depth understanding of the specific antiviral mechanisms of these LLPS enhancers. This potentiation may inhibit viral replication by interfering with the unwinding process of viral genomic RNA (gRNA) or by inducing premature assembly of aberrant ribonucleoprotein (RNP) complexes [48].

Nucleoside analogues are a class of compounds that mimic the structure and function of nucleotides. It has been found that certain nucleoside analogues can interfere with the interaction between nuclear proteins and RNA, thereby inhibiting LLPS formation. Adenosine triphosphate (ATP) can competitively bind specifically to the RNA-binding domain (RBD) of N proteins, thereby modulating the LLPS of the SARS-CoV-2 N protein. ATP binds not only to nucleic acids but also to arginine residues in the proteins, competitively dislodging the nucleic acids from the proteins and solubilizing the LLPS. S2m (derived from the 32-polymer stem-loop II of the SARS-CoV-2) and A24 (a 24-oligomer non-specific nucleic acid) can modulate LLPS through dynamic and multivalent interactions with arginine/lysine residues in the structural domains and disordered regions of proteins [49]. Moreover, a single-stranded DNA aptamer (N-Apt17) and its circular bivalent form (cb-N-Apt17) can effectively disrupt the LLPS of the SARS-CoV-2 N protein, which presents a potential inhibitory ability on viral replication [58].

Interference with the dimerization/oligomerization of N proteins regulates LLPS. The C-terminal structural domain (CTD) of N proteins is responsible for the dimerization/oligomerization to form higher-order structures, which is essential for the LLPS process. Therefore, designing compounds that interfere with the dimerization/oligomerization of the CTD of the N protein may help to prevent the beginning of LLPS and thus inhibit viral replication and transmission. It was found that an oligomerization site exists in the disordered LKR of the CoV N protein. This site contributes to LLPS and is essential for the assembly of high-level protein–nucleic acid complexes. The site is formed through trimeric coiled-coil interactions of a transient helical structure, and key amino acid residues stabilize hydrophobic and electrostatic interactions between adjacent helices. This oligomerized structural domain is highly conserved in the viable SARS-CoV-2 genome and conserved in related coronaviruses, making it a potential target for antiviral therapy [39].

SG is a cytoplasmic RNA protein aggregate induced by viral infection. By interfering with the association of the N protein with SG, it may be possible to block the ability of viruses to use SG to evade the immune response of host cells, thereby preventing viral replication and transmission in host cells. Imatinib and decitabine were identified as potential modulators of G3BP1/2 genes and regulators, suggesting that they may serve as therapeutic agents for COVID-19. Molecular docking analyses revealed that the binding affinity of imatinib and decitabine to G3BP1/2 was significantly higher than their affinity to the SARS-CoV-2 N protein. Therefore, imatinib and decitabine could be candidates for targeting both N and G3BP1/2 proteins [59]. In addition, it has been shown that the RNA degrader RNase A can completely block the LLPS properties of NCAP and its association with SG [60]. Moreover, HCoV N protein homologs can directly bind to the low-complexity domain of FUS, an SG-containing protein, to disrupt SG homeostasis via accelerating its liquid-to-solid phase transition and amyloid aggregation [61]. Moreover, FOXA1, YY1, SYK, E2F-1, and TGFBR2 have been identified as activators of the G3BP1/2 genes, and SIN3A, SRF, and AKT-1 have been identified as their repressors [59]. SARS-CoV N proteins can specifically interact with the immunomodulatory factors YTHDF3, USP10, and PKR [62]. Hence, the combination of these activators and repressors of SG and immunomodulatory factors was then used to screen for drugs that could alter their gene expression profile; this may enable the possibility of modulating the immune response and enhance the body’s resistance to viruses.

## 7. Summary and Outlook

CoVs are the most important human and animal pathogens, and they seriously threaten public health safety. However, there are currently no specific drugs available for the treatment of CoVs. CoVs exhibit a high degree of complexity in their pathogenesis, and a large number of unanswered questions remain in current scientific research. Given the long lead time, high cost, and multiple scientific and technical challenges in the development of a single antiviral drug, the vaccination strategy remains the current optimal choice for the prevention and control of coronavirus infections due to its relatively mature development process and proven safety record. Despite this, drug therapy plays a crucial role among infected persons. The medication provides an individualized treatment plan that is tailored to the patient’s specific condition, physical differences, and age profile, which significantly improves the effectiveness of the treatment. The effectiveness of the vaccine may be challenging given the mutating nature of CoVs. However, drug therapy has demonstrated the potential to cope with different viral variants by flexibly adjusting the drug type and dose. Furthermore, the proposed therapeutic strategy based on the liquid–liquid phase separation mechanism of CoVs may have a broad spectrum of applicability, not only to the current virus but also to other viruses that possess the liquid–liquid phase separation mechanism. The proposed therapeutic strategy provides a new perspective for drug discovery and development, which is important for the future development of antiviral drugs.

LLPS is still a new field for exploring viral replication and participation in the regulation of host biological processes. In recent years, studies have proven that the process of SARS-CoV-2-induced LLPS involves the interaction of N proteins and their Nsps with RNA, as well as the regulation of temperature, pH, salt concentration, and ATP. On the one hand, CoV-induced LLPS is beneficial to the aggregation of viral replication complexes, which can improve the efficiency of viral replication and assembly and promote virus proliferation in cells. On the other hand, CoV-induced LLPS can isolate host antiviral factors and then escape the host immune response, especially host innate immunity. Based on this understanding, a variety of potential drugs and therapeutic strategies for COVID-19 have been explored to inhibit the proliferation of SARS-CoV-2. However, there is some variability observed among different CoVs, and it remains unclear whether other CoVs exhibit the same mechanism to induce LLPS.

Although there have been some developments in our understanding of the mechanism and function of coronavirus-induced LLPS in recent years, it is worth noting that most studies are descriptive results from in vitro assays. Therefore, future studies will need more established tools to focus on a better mechanistic understanding in vivo. For example, the design of proteins specifically labeled with viral components for real-time observation in cells will provide a basis to further understand the process of LLPS initiation under natural coronavirus infection conditions and provide a basis for the design of intervention strategies.

The molecular mechanism through which coronaviruses evade the host’s antiviral innate immunity through LLPS is currently a research hotspot in virology. CoV N proteins are the most synthesized proteins in virus-infected host cells. They play a crucial role in viral replication and assembly and are extensively involved in regulating host cell biological processes [43]. Several CoV N proteins have been confirmed to inhibit the host innate immunity in cells. Due to the high degree of variability in CoVs, differences between viral strains may impact their ability to evade host immunity. This needs further investigation. Moreover, there is still limited understanding of the molecular mechanisms through which LLPS influences the host antiviral innate immune response. In the future, researchers can employ a variety of strategies to address the aforementioned issues. For example, high-throughput screening and gene editing technologies are being used to identify and validate the key factors involved in LLPS and their mechanisms of action. Researchers should conduct collaborative, interdisciplinary studies to integrate knowledge from biology, chemistry, physics, and other fields to fully understand the molecular mechanisms through which coronaviruses evade the immune system.
